# From deficiency to toxicity: Magnesium increases cannabinoid and terpene production in cannabis plants

**DOI:** 10.1186/s42238-025-00358-9

**Published:** 2025-12-10

**Authors:** Dalit Morad, Nirit Bernstein

**Affiliations:** Institute of Soil Water & Environmental Sciences, Volcani Institute, P.O.B 15159, 68 HaMaccabim Road, Rishon LeZion, 7505101 Israel

## Abstract

**Background:**

The biological-therapeutic activity of medical cannabis is based on a wide range of secondary metabolites, including terpenes and the cannabis-specific cannabinoids, which are produced to the highest concentrations in the plants’ unfertilized inflorescences. Recent evidence demonstrate effects of exogenous factors on cannabinoid and terpene profiles in cannabis, with substantial impact to the macronutrients N,P,K. However, knowledge on effects of most other essential mineral-nutrients, including magnesium (Mg), is lacking. Mg-availability is of potential relevance for the pharmaceutical-profile of cannabis, as it is required for activity of key enzymes in the biosynthesis pathway of cannabinoids and terpenes, and for the formation of Geranyl-diphosphate (GPP) that involves in the biosynthesis of both specialized secondary-metabolite groups. The present study thus evaluated the hypothesis that Mg-supply affects cannabinoid and terpene biosynthesis in cannabis, in addition to its effect on plant-function, and in relation to induced-changes to the plant-organs ionome.

**Methods:**

To test the hypothesis, cannabis plants were grown under five Mg levels ranging 2–140 mg L^−1^ (ppm), and morpho-physiology analyses, cannabinoid and terpene profiles of inflorescences from two developmental-orders, and ionome-profiling of the plant-organs were conducted. The wide concentration rage studied was aimed to pinpoint the optimal supply concentrations for plant morpho-physiology and secondary-metabolite production, and the deficiency and toxicity concentration thresholds for the reproductive stage.

**Results:**

The results revealed high sensitivity of cannabinoid and terpene biosynthesis to Mg, thus supporting the hypothesis. Morpho-physiological function, and concentrations of the specialized metabolites were restricted by low Mg-availability of 2–20 mg L^−1^, and were optimal under the supply treatment of 35 mg L^−1^ Mg. While under over-supply levels of 70–140 mg L^−1^ Mg, which impacted physiological-function and reduced reproductive biomass by 12.5%, secondary-metabolism remained unharmed.

**Conclusions:**

Taken together, the results reveal that 35 mg L^−1^ Mg is within the optimal range for excelled yield quantity as well as quality, i.e., high secondary metabolite production, with suppression of cannabinoid and terpene production under lower or higher Mg supply.

**Supplementary Information:**

The online version contains supplementary material available at 10.1186/s42238-025-00358-9.

## Introduction

The cannabis plant is employed by humanity as a medicinal plant since ancient times (Andre et al. 2016). The biological activity of this plant is based on a large number of bio-active secondary metabolites, including flavonoids, terpenoids, and the cannabis-specific cannabinoids (Radwan et al. 2021). These secondary metabolites are produced and accumulate in glandular trichomes that develop to the highest densities in female inflorescences. Cannabinoids, are terpenophenolic compounds that are formed in the cannabis plant in acidic forms that contain a carboxyl group, which can decarboxylate in the plant and post-harvest to the neutral forms that are the biologically active compounds. Cannabigerolic acid (CBGA) the precursor of the three main cannabinoids Δ^9^-tetrahydrocannabinolic acid (Δ^9^-THCA), Cannabidiolic acid (CBDA) and cannabichromenic acid (CBCA) is produced from olivetolic acid and the mevalonate-pathway intermediate geranyl pyrophosphate (GPP) by geranyl pyrophosphate: olivetolate geranyltransferase (GOT) (Luo et al. 2019; Nahar et al. 2021). Most other cannabinoids are produced from these three main cannabinoids. The remaining cannabinoids are minor cannabinoids originating from GPP and Divarinic acid that are formed from the precursor cannabigerovarinic acid (CBGVA) and the three main cannabinoids that are formed from it are Δ^9^-tetrahydrocannabivarinic acid (THCVA), cannabidivarinic acid (CBDVA) and cannabichrovarinic acid (CBCVA) (Tahir et al. 2021). As in other plants, the terpenoids in cannabis are derived from the cytosolic mevalonate pathway, or from the plastidial Deoxyxylulose phosphate/methyl-erythritol phosphate (DOXP/MEP) pathway, from isopentenyl diphosphate (IPP) and its allylic isomer dimethylallyl diphosphate (DMAPP). IPP and DMAPP are used by prenyl transferases in head-to-tail condensation reactions to produce geranyl diphosphate, Farnesyl diphosphate and geranylgeranyl diphosphate, the immediate precursors of the monoterpenes, sesquiterpenes and diterpenes, respectively (Cheng et al. 2007; Flores-Sanchez and Verpoorte 2008).

It is well documented for many plant species that a wide array of environmental conditions and constrains including, salinity, drought stress, low and high temperature and mineral nutrition, can impact secondary metabolite biosynthesis (Ramakrishna and Ravishankar 2011; Bernstein et al. 2019; Li et al. 2020). In cannabis as well, a growing body of evidence demonstrates that the secondary metabolite profile is determined by an interaction of genetic and environmental factors (Gorelick and Bernstein 2014). Inheritable genetic factors determine basic chemotypic differences between varieties, organs, and position in the plant (Danziger and Bernstein 2021b; Shiponi and Bernstein 2021b); and recent studies demonstrated a considerable impact of environmental condition on the cannabinoid and terpene profiles in the cannabis plant, including mineral nutrition (Saloner and Bernstein 2021, 2022a, 2022b; Shiponi and Bernstein 2021b; Bassolino et al. 2023; Llewellyn et al. 2023), light (Eichhorn Bilodeau et al. 2019; Danziger and Bernstein 2021a; Sae-Tang et al. 2024), temperature (Holweg et al. 2025), salinity (Yep et al. 2020), and drought (Caplan et al. 2019). The interest in the cannabis phytochemicals sources from their substantial therapeutic potential for modern medicine. Due to legal restrictions at the last decades, research on the cannabis plant and its phytochemicals was restricted globally bringing on the need to close the knowledge-gap concerning the response of the medical cannabis plant and its chemical profile to exogenous factors and environmental conditions.

Mineral nutrients are among the main factors that affect plant development, function, and metabolism (Marschner and Rengel 2011; Shiponi and Bernstein 2021b; Westmoreland and Bugbee 2022) and optimal growth, production and plant function require sufficient availability of all the essential plant macro and micronutrients. A growing body of information concerning the requirements of the drug-type (medical) cannabis plant is available for major macronutrients including nitrogen (Saloner and Bernstein 2021, 2022b); phosphorous (Shiponi and Bernstein 2021b, Silva et al. 2019; Westmoreland and Bugbee 2022), potassium (Saloner and Bernstein 2022a), as well as interaction between them (Bevan et al. 2021, Kpai et al. 2024). However, information about the response and requirements of ‘drug-type’ cannabis for most other essential mineral nutrients, including magnesium (Mg), is sparce. At the vegetative stage, development and function of cannabis ‘drug-type’ plants was reported optimal under Mg supply of 35–70 mg L^−1^ (Morad and Bernstein 2023), and similarly in Hemp 50 and 75 mg L^−1^ Mg were reported optimal under both the vegetative and the reproductive stages of development (Veazie et al. 2021).

Magnesium is an essential macronutrient required for plant development and function due to its fundamental role in key processes in the plant, including photosynthesis, synthesis of nucleic acids and proteins, energy metabolism, and carbohydrate translocation (Chen et al. 2018). Mg availability was demonstrated to affect production of secondary metabolites in numerous plants including tea (ILi et al. 2021), *Tanacetum parthenium* (Farzadfar et al. 2017), and apple (Zahedzadeh et al. 2015); and it is well known to affect chlorophyll synthesis and degradation and thereby photosynthesis (Kobayashi and Tanoi 2015; Yang et al. 2023). Of potential relevance for cannabis quality, Mg is required also for the activity of several key enzymes in the biosynthesis pathway of cannabinoids and terpenes, the two major groups of biologically active secondary metabolites in cannabis, including GOT, hexanoyl-CoA synthetase, and1-Deoxy-D-xylulose 5-phosphate synthase (Eisenreich et al. 2001; Degenhardt et al. 2017; Gräwert et al. 2011), as well as for the formation of Geranyl diphosphate (GPP) that participates in the biosynthesis of both terpenes and cannabinoids (Degenhardt et al. 2017).

The present study thus aimed to evaluate the impact of Mg supply on cannabis plants at the reproductive growth phase. The hypothesis guiding the workplan was that Mg availability affects development, function, and secondary metabolite production in the plants, and in relation to induced changes to the ionome of the plant organs. To test this hypothesis, we studied developmental, biochemical, and physiological responses of the plants to Mg supply ranging from 2 to 140 mg L^−1^ Mg, and profiled the ionome of the plant organs. Understanding effects of Mg on the cannabis plants at the reproductive stage of development is important for regulation of the bioactive metabolites profile in the cannabis inflorescence yield. The range of Mg concentrations tested, aimed to reveal the optimal supply rates for Mg by covering inputs ranging from deficiency to toxicity concentrations. Directing cultivation practices for production of yield suitable for the medical industry requires understanding of the interrelation between secondary metabolism and plant function.

## Materials and methods

### Plant material and growing conditions

‘Anafurna’ (Canndoc LTD, Israel)*,* a medical drug-type cannabis (*Cannabis sativa* L.) cultivar, was used for the study. It contains similar THC and CBD concentrations (~ 7%), and has an indica-like morphology. The plants were propagated in vermiculite by cloning from a single mother plant (Hydroisrael, Rishon LeZion, Israel). Thirty days after dissection from the mother plants, rooted cuttings selected for uniformity were planted in a perlite growing medium containing 100% perlite, of the size range 0.075–2.5 mm, with a specific weight of 80–90 g per liter (212, Agarkel, Bonim, Israel) in three-liter pots. For the vegetative growth phase, the plants were grown under a long photoperiod (18/6 h light/dark), at 25 °C, in a controlled growing room, and were irradiated at 400 μmol m^−2^ s^−1^ (metal halide lamps; Solis Tek Inc, Carson, CA; 25.9 mol m^−2^ s^−1^). The level of Mg supplied at the vegetative growth phase was 35 mg L^−1^ Mg which was identified in a previous study to be within the optimal level for cannabis plants growth and function (Morad and Bernstein 2023). After one week of adjustment to these conditions followed by 16 days of vegetative growth, the plants were divided into five treatment groups of increasing Mg concentrations: 2, 20, 35, 70 and 140 mg L^−1^, (ppm), (e.g., 0.08, 0.82, 1.44, 2.88, and 5.76 mM Mg), with six replicated plants per treatment group, and short photoperiod (12/12 h light/dark) was applied using High Pressure Sodium bulbs (980 μmol m^−2^s^−1^, Greenlab by Hydrogarden, Petah Tikva, Israel). The differential Mg treatments were applied until the termination of the experiment and harvest at maturation.

The plants were irrigated daily with 1 L h^−1^ discharge-regulated drippers (Netafim, Tel-Aviv, Israel), 1 dripper per pot. In each irrigation event the volume of irrigation was 330–500 ml/pot/day, adjusted to generate 30% drainage. The plants were cultivated under the differential Mg treatments under short day for 59 days until the end of the experiment at maturation. Weekly analyses of the fertigation solutions confirmed that Mg concentrations were in accord with the planed treatment concentrations. pH of the irrigation solution of all treatments was adjusted to 5.8.

Fertilizers were supplied dissolved in the irrigation solution at each irrigation. The composition of the fertigation solution was (in mg L^−1^): 160 N (14 N-NH_4_^+^, 146 N-NO_3_^−^), 175 potassium (K) and 30 phosphorous (P), which are the optimal concentrations we identified in previous studies for cannabis (Saloner and Bernstein 2021, 2022a; Shiponi and Bernstein 2021b), with 110 calcium (Ca^2+^), 85 S-SO_4_^−2^, 1.7 iron (Fe^2+^), 0.8 manganese (Mn^2+^), 0.10 boron (B^3+^), 0.4 zinc (Zn^2+^), 0.04 cupper (Cu^2+^), 0.03 molybdenum (Mo^2+^), and 85 S-SO_4_ in the 4 lower Mg treatments and 144 S-SO_4_^2−^ in the highest Mg treatment. SO_4_ was chosen as the accompanying ion for a fraction of the highest Mg treatment because we found in a preliminary measurement that uptake of S by cannabis plants does not elevate with elevated supply at the concentration range used (unpublished data). The fertigation solutions were prepared from Mono-ammonium phosphate (MAP), Mono-potassium phosphate (MKP), calcium nitrate, potassium nitrate, magnesium nitrate, magnesium sulfate, and sulfuric acid. Cu, Mn, Mo, Zn were introduced chelated with Ethylenediaminetetraacetic acid (EDTA), Fe as chelated with Ethylenediamine-N,N′-di[(2-hydroxy-5-sulfophenyl)acetic acid] (EDDHSA) (Barkoret, ICL, Haifa, Israel), and B as B-7000 (ICL, Tel-Aviv, Israel). The experiment had a complete randomized design, with six replicates per treatment. All measurements were accordingly conducted with 5–6 replications. Light intensity was monitored weekly at 50 cm^2^ interval throughout the growing room and adjusted to a maximum of ± 5% variability. To maximize reflection and light uniformity the growing room walls were coated with a reflective material.

### Biomass accumulation and plant architecture and development

The number of nodes on the main stem and stem-diameter were measured at the termination of the experiment, on day 58 after the beginning of the Mg treatments and the switch to the short-day photoperiod. Stem-diameter was measured with an electronic caliper (YT-7201, Signet Tool International Co., Ltd., Shengang, Taiwan) 3 cm from the plant base.

Plant height was measured weekly (nine times), as the distance from the top of the main stem to the base of the plant. Biomass of roots, stems, leaves, inflorescence leaves and inflorescences were measured at the end of the experiment, 59 days after the initiation of the Mg treatments. Dry weights were measured following desiccation for 72 h under 64°C.

### Physiological parameters

The plant tissue was sampled for physiology analysis on day 37 after the switch to the short photoperiod and the initialization of the Mg fertilization treatments.

#### Membrane leakage, osmotic potential and relative water content

Membrane leakage as an indicator of stress level of the plant tissue, and osmotic potential as an indicator of water relations of the tissue, were analyzed in fan leaves at two developmental stages. The analyzed leaves were the youngest-mature leaf on the main stem (from the second node from the top of the plant) [Young-mature leaf], and an old-mature leaf (from the fifth node from the top of the main stem). Following removal from the plant, the leaves were washed twice with distilled water. For membrane leakage analysis, a leaflet of the sampled leaves was placed in a test tube containing 30 ml of distilled water and the analyses was conducted following (Saloner et al. 2019). Two leaflets from each sampled fan leaf were used for osmotic potential determination. They were frozen in −80ºC in 1.7 ml Eppendorf test tube and analyzed as following Saloner and Bernstein (2020).

For relative water content (RWC) analysis the sampled leaves were weighed immediately after detachment from the plant and immersed in test tubes containing 50 ml of distilled water. Measurements and calculations were conducted following (Bernstein et al. 2010).

#### Photosynthetic pigments and gas-exchange parameters

For the analysis of the concentration of photosynthetic pigments, five leaf discs, 6 mm in diameter were taken from the central leaflet of each the youngest mature leaf, and a mature leaf located on the main stem. The sampled leaf discs were placed into 2 ml test tubes containing 80% ethanol (0.8 ml), and frozen at −20°C until analysis. Pigment extraction from the tissue, and analysis, were performed following Shiponi and Bernstein (2021a).

Stomatal conductance, photosynthesis and transpiration rate, and intercellular CO_2_ concentration were measured on two leaves: the youngest mature fan leaf [Young-mature leaf] and an old-mature leaf located on the second and the fifth node from the top of the main stem, respectively. The measurements were conducted with a Licor system (6400 XT, LI-COR, Lincoln, NE, United States), 38 days after the switch to the short photoperiod and the initiation of the Mg treatments. WUEi (Intrinsic water use efficiency) that is the response of WUE at the leaf level was computed by dividing the net photosynthetic results by the stomatal conductance. The measurements were conducted on six replicated plants.

### Cannabinoids and terpenoids analyses

Cannabinoid concentrations were analyzed in inflorescences from two locations in each plant; the apical inflorescence of the main stem [primary inflorescence] and the apical inflorescence of the lowest branch of the main stem [secondary inflorescence]; and terpenoid concentrations were analyzed in the primary inflorescence, 59 days following the switch to the short photoperiod when 30% of the glandular stalked-trichome heads were of amber color. The inflorescences were hand-trimmed, i.e., the protruding parts of the inflorescence-leaves were removed from the inflorescence. The trimmed inflorescences were dried at 19.5º C and 45% relative humidity on drying trays in the dark, in an environment-controlled chamber. After 14 days of drying when the inflorescences had 10% moisture-content, the samples were transitioned to curing in the dark at 25Cº for two months prior to analyses.

#### Terpenoid analyses

Terpenoid concentrations were analyzed by Gas Chromatography-Mass Spectrometry (GCMS) as is detailed by Saloner et al. (2021). In short, 100 mg of dried plant material was ground in liquid N_2_ to a fine powder. Volatiles were extracted with 2 mL MTBE (methyl tertiary-butyl ether), containing 100 ppm of ethyl myristate as an internal standard. The upper MTBE layer was separated and dried with Na_2_SO_4_ and the analysis of terpenoids was performed as is detailed by Saloner et al. (2021). A 1 μL aliquot of the sample was injected into a GC-MSD system (model 6890 N/5973 N, Agilent Technologies CA, USA) equipped with Rxi-5sil ms column (30 m length × 0.25 mm i.d., 0.25 μm film thickness, stationary phase 95% dimethyl- 5% diphenyl polysiloxane), using helium (11.18 psi) as a carrier gas with splitless injection. Compounds identification was conducted by comparing the mass spectra and relative retention indices with those of authentic samples or with those found in the literature and supplemented with W10N11 and QuadLib 2205 GC–MS libraries.

#### Cannabinoid analyses

Cannabinoid analysis was conducted following Saloner et al. (2021). In short, 50 mg of ground plant material was extracted with 10 ml of 100% analytical ethanol and was shaken for 1 h at room temperature. One ml of the extract was filtered with a 0.22 µm pore size polyvinylidene difluoride (PVDF) membrane filter (Bar-Naor ltd, Ramat Gan, Israel). Cannabinoid concentrations in the filtered plant extracts were analyzed by HPLC (Jasco 2000 plus series) which consisted of a quaternary pump, an autosampler, a column compartment, and a PDA detector (Jasco, Tokyo, Japan). The detection was carried out in a spectrum mode, at the wavelength range 200–650 nm. Chromatographic separations were performed with a 3 µm Polar C18 column Luna Omega (Phenomenex, Torrance, CA USA) using acetonitrile:water 75:25 (v/v) with 0.1% (v/v) formic acid, in isocratic mode, with a 1.0 mL/minute flow rate. Quantifications were based on analytical standards: CBCA, THCVA, CBN, CBT, CBDA, CBC, CBDVA, CBGA (Sigma-Aldrich, Germany) and Δ^9^-THCA (THCA-A), Δ^9^-THC, CBD, CBDA (Restek, Pennsylvania, USA).

Total concentrations of THC, CBD, and CBC—the three main cannabinoids, i.e., the sum of the acidic and neutral form of each of these cannabinoids were calculated taking into account differences in mass of the carboxylated vs. decarboxilated forms as of the following equations:

$$\text{Total THC }=\text{ THCA}\times 0.877+\text{THC}$$

$$\text{Total CBD }=\text{ CBDA}\times 0.877+\text{CBD}$$

$$\text{Total CBC }=\text{ CBCA}\times 0.877+\text{CBC}$$

### Inorganic mineral analysis

Concentrations of inorganic nutrients in the plant organs were analyzed at the end of the experiment, 9 weeks after the initiation of the Mg treatments (e.g., 59 days). Plants were separated into leaves, stems, roots, inflorescence-leaves and inflorescences. The shoot organs were rinsed twice with distilled water and blotted dry, and following removal from the growing media the roots were gently rinsed three times in distilled water and blotted dry. Fresh weights were measured with a digital balance (Precisa 40SM-200A, Zurich, Switzerland). Dry biomass was measured after drying at 64ºC for 48 h. For the chemical analysis, the dry tissue samples were each ground to a powder and analyzed for concentrations of N, P, K, Ca, Mg, Fe, Mn, Cu and Zn. Two procedures were used for extraction of the mineral elements from the plant material. For Ca, Mg, Mn, Zn and Fe analysis, the ground tissue was heat- digested with HNO_3_ (65%) and HClO_4_ (70%) (Bernstein et al. 2005), and the elements were analyzed with a dual-view High-Resolution ICP-OES spectrometer PlasmaQuant PQ9000 (Analytik Jena GmbH Co. KG, Jena, Germany). For N, P and K analysis, the ground tissue was heat-digested with H_2_SO_4_ (98%) and H_2_O_2_ (70–72%). P and N were analyzed with Autoanalyzer (Lachat Instruments, Milwaukee, WI, USA), and K by Flame Photometery (410 Flame Photometer Range, Sherwood Scientific Ltd., The Paddocks, UK) (Morad and Bernstein 2023). Electrical conductivity (EC) and pH in the irrigation and drainage solutions were analyzed throughout the experiment, by an EC meter (A212 Orion Star, Thermo Fisher Scientific, Waltham Massachusetts, USA), and a pH meter (1500, Eutech Instruments, Thermo Fisher Scientific, Waltham Massachusetts, USA).

### Statistical analyses

The data were analysed by one-way or two-way ANOVA followed by Tukey HSD post-hoc test for separation of means. The data met the assumptions of normality and homogeneity of variances. Comparison of relevant means was performed by Fisher’s LSD test at 5% level of significance. The analysis was done with Jmp software (ver. 14, SAS 2018, Cary, NC, USA).

## Results

### Plant growth and development and visual appearance

**Fig. 1:**
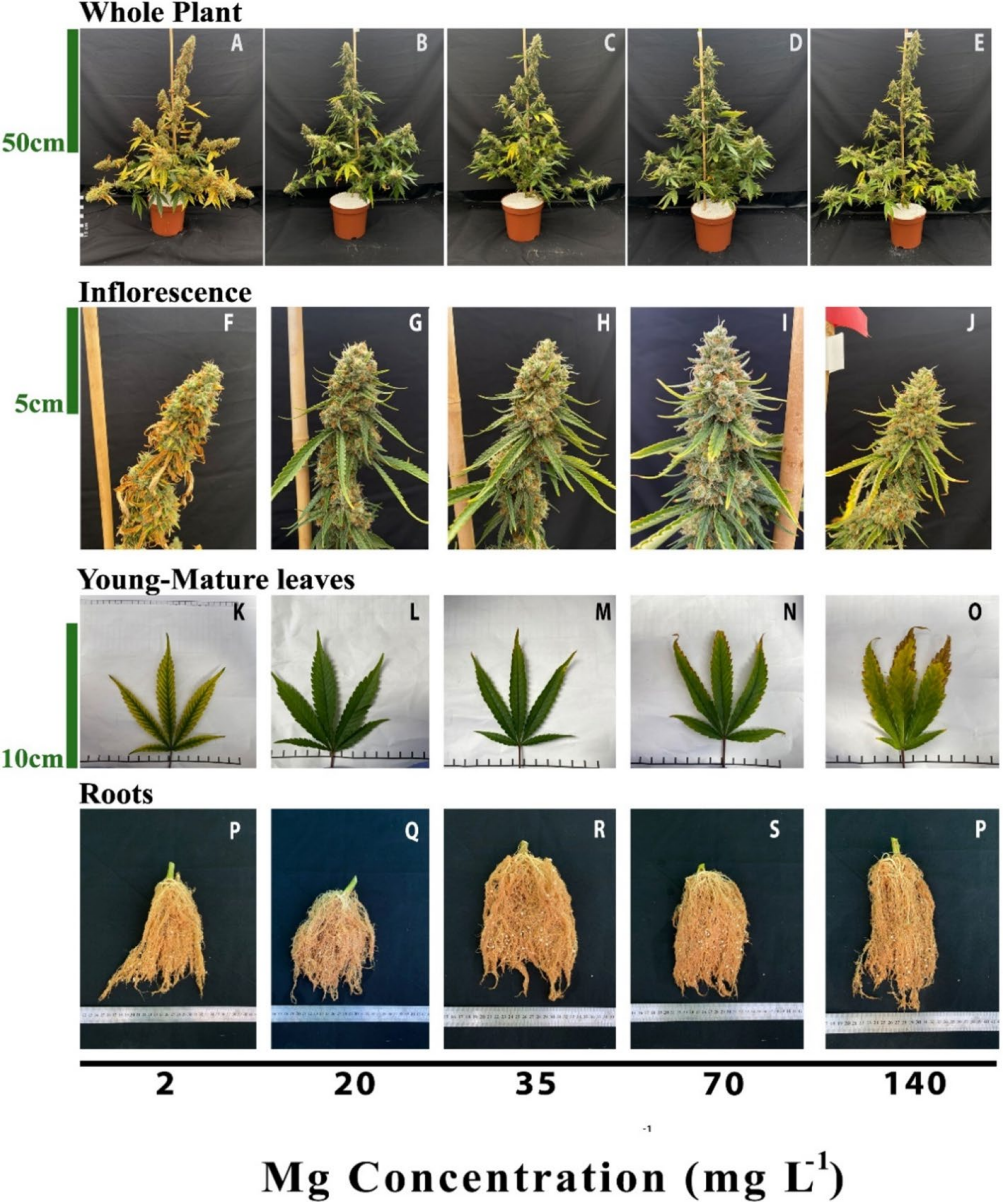
Appearance of the plants (top row **A**-**E**), inflorescence (second row **F**-**J**), young-mature leaves (third row **K**–**O**) and roots (bottom row **P**–**T**) under increasing Mg supply. Plants were cultivated under 2, 20, 35, 70, 140 mg L^−1^ Mg. Young leaves images are of the youngest, fully developed leaf on the main stem. Images of leaves, inflorescences and whole plants were taken 45 days after the initiation of the Mg fertigation treatments. Roots images were taken 59 days after the initiation of the Mg fertigation treatments

**Fig. 2:**
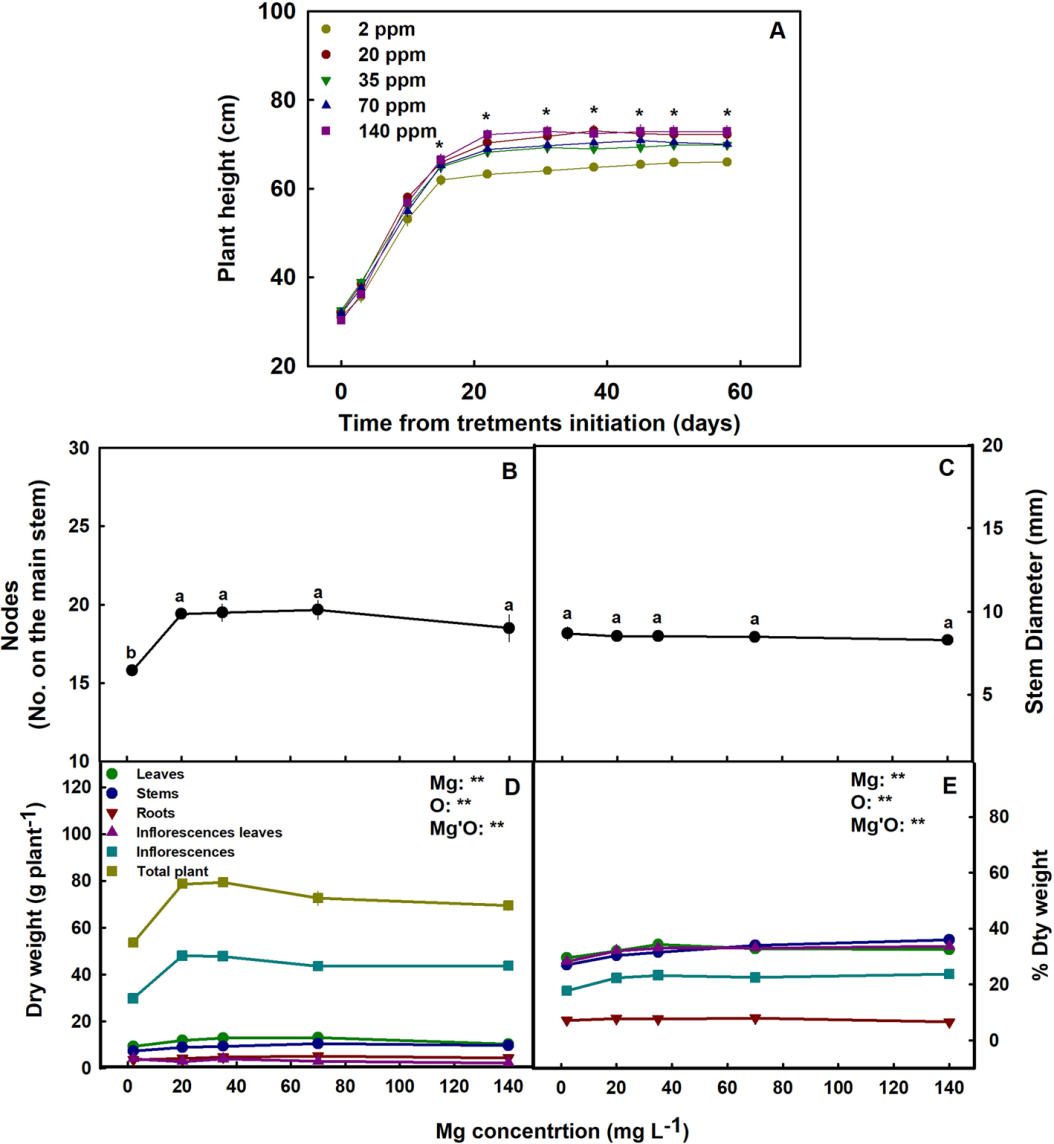
Effect of Mg supply on the development of medical cannabis plants at the reproductive growth phase. Plant height (**A**), number of nodes on the main stem (**B**), stem diameter (**C**), dry weight per plant (**D**), and percentage of dry weight in the plant organs and the entire plant (**E**). Presented data are averages ± SE (*n* = 6). In **A** Asterisk above the means represent significant differences between the Mg treatments for a given day by Tukey HSD test (*α* = 0.05). In **B** and **C** different letters above the means represent significant differences by Tukey HSD test at *α* = 0.05. In **D** and **E** results of two-way ANOVA indicated as ** *p* < 0.05, *F*-test; NS, not significant *p* > 0.05, *F*-test. In the ANOVA results Mg is magnesium, O is organ, and Mg’O represents the interaction between Mg and O

The visual appearance of the plant organs is indicative of the plant response to the Mg dosage (Fig. 1). The plants supplied with 2 mg L^−1^ Mg developed interveinal chlorosis in leaves, and small inflorescences with scorched and dead inflorescences leaves (Fig. 1F, K), which resulted in lowest inflorescence biomass (Fig. 2D) and an overall appearance of chlorotic plants (Fig. 1A). This deficiency symptoms were especially prominent in leaves and inflorescence leaves from the upper part of branched, while leaves and small inflorescences from the bottom of the plants remained green and less harmed (Fig. 1A, F, K).

Under higher Mg concentrations (20–70 mg L^−1^) the plants looked similar and undamaged (Fig. 1B-D), except for the appearance of toxicity symptoms under 70 mg L^−1^ Mg, e.g., burnt leaf tips followed by leaf chlorosis. Under the highest Mg supply of 140 mg L^−1^ the plants suffered the same toxicity symptoms, which were more severe, and developed smaller inflorescences with slightly burnt inflorescence-leaves tips (Fig. 1E, J, O). There was no difference in the appearance of the roots between treatments (Fig. 1P-T).

The Mg treatments affected plant development. The plants that received 2 mg L^−1^ Mg were significantly shorter and less branched then the 70–140 mg L^−1^ plants (Fig. 2 A-B) and had the lower plant biomass (Fig. 2D) and percentage of dry weight in the shoot organs (Fig. 2E). Inflorescence biomass in the 70–140 mg L^−1^ treatments was 43–60% higher compared to the 2 mg L^−1^ Mg treatment. The diameter of the stem was not affected by the Mg treatments (Fig. 2C).

### Membrane leakage, osmotic potential, and Relative water content

**Fig. 3:**
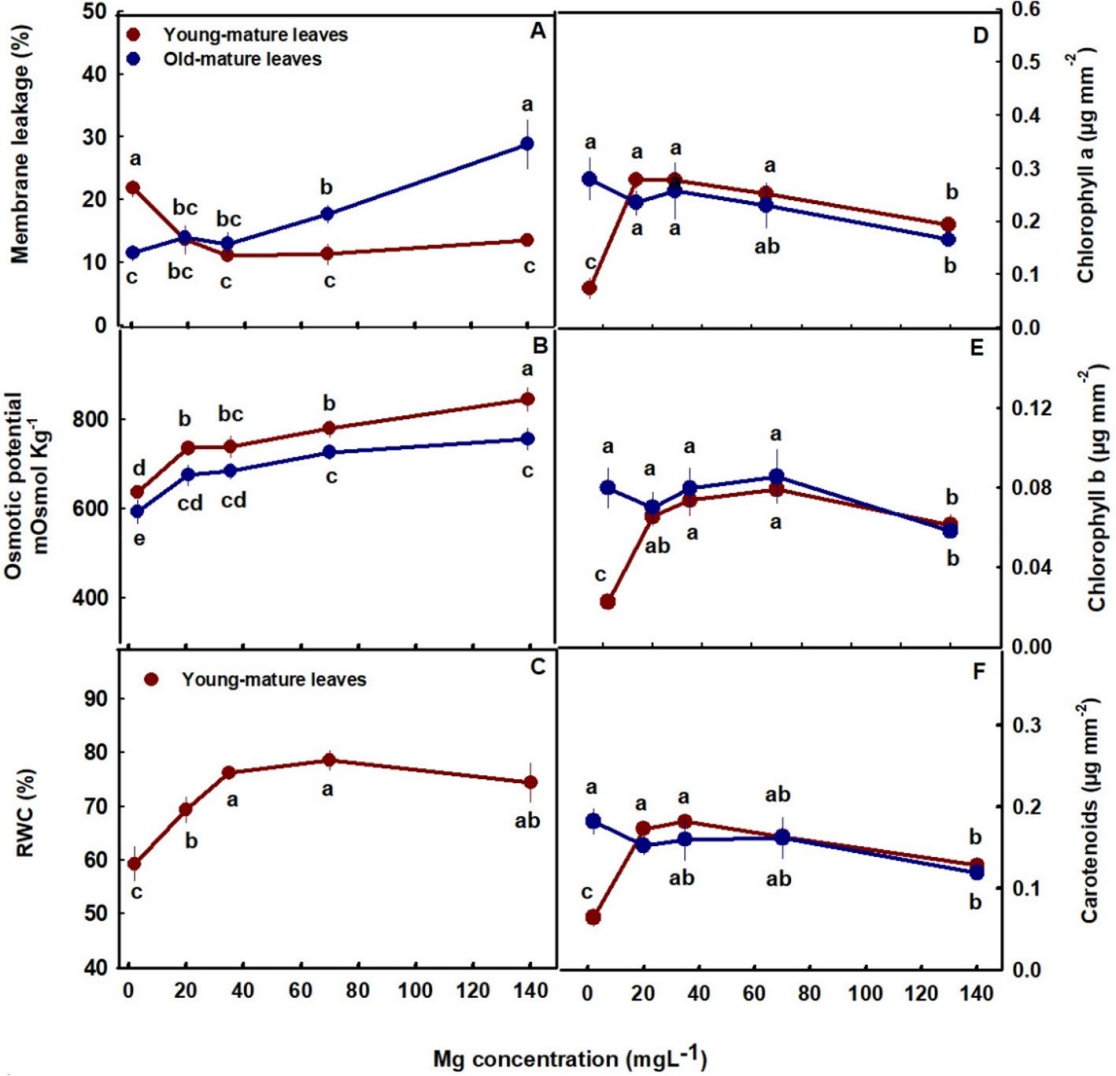
Effect of Mg application on physiological characteristics and photosynthetic pigments of medical cannabis plants at the reproductive growth phase. Membrane leakage (**A**), Osmotic potential (**B**), Relative water content (RWC) (**C**), Chlorophyll a (**D**), Chlorophyll b (**E**), and carotenoids (**E**) in young-mature and old-mature leaves. RWC is presented only for young-mature leaves. Presented data are averages ± SE (*n* = 6). Different small letters above the means represent significant differences between treatments by Tukey HSD test at *α* = 0.05

Membrane leakage, an indicator of tissue stress, was highest in the young-mature leaves under limited Mg supply (2 mg L^−1^ Mg) and decreased with the increased in Mg up to the concentration of 20 mg L^−1^ Mg and then stabilized. This shows sensitivity of plants tissues to Mg deficiencies in young-mature leaves. In old-mature leaves membrane leakage was stable at the low concentration range of 2–35 mg L^−^1 Mg but sharply elevated with further increase in Mg supply (Fig. 3A), which demonstrates sensitivity to high concentration (70–140 mg L^−1^ Mg). This is in accord with the results we obtained for the vegetative growth phase (Morad and Bernstein 2023). Osmotic potential increased with the increase of Mg supply in both young-mature and old-mature leaves and was higher in young-mature than old-mature leaves (Fig. 3B). RWC in young-mature leaves increased up to the concentration of 35 mg L^−1^ Mg (Fig. 3C).

### Photosynthetic pigments, photosynthesis, and gas exchange parameters

The photosynthetic pigments tested, chlorophyll a, chlorophyll b, and carotenoids showed a similar response trend to the level of Mg imposed on the plants (Fig. 3D-F). In young-mature leaves the pigments concentration demonstrated optimum response curves, with lowest concentrations under the deficiency concentration (2 mg L^−1^ Mg), higher concentrations under 20–70 mg L^−1^ Mg and a decrease under the highest Mg concentration (140 mg L^−1^ Mg). In contrast to young-mature leaves, in old-mature leaves the concentration of the three photosynthetic pigments was highest under low Mg supply and reduced under the toxicity concentration (Fig. 3D-F).

**Fig. 4:**
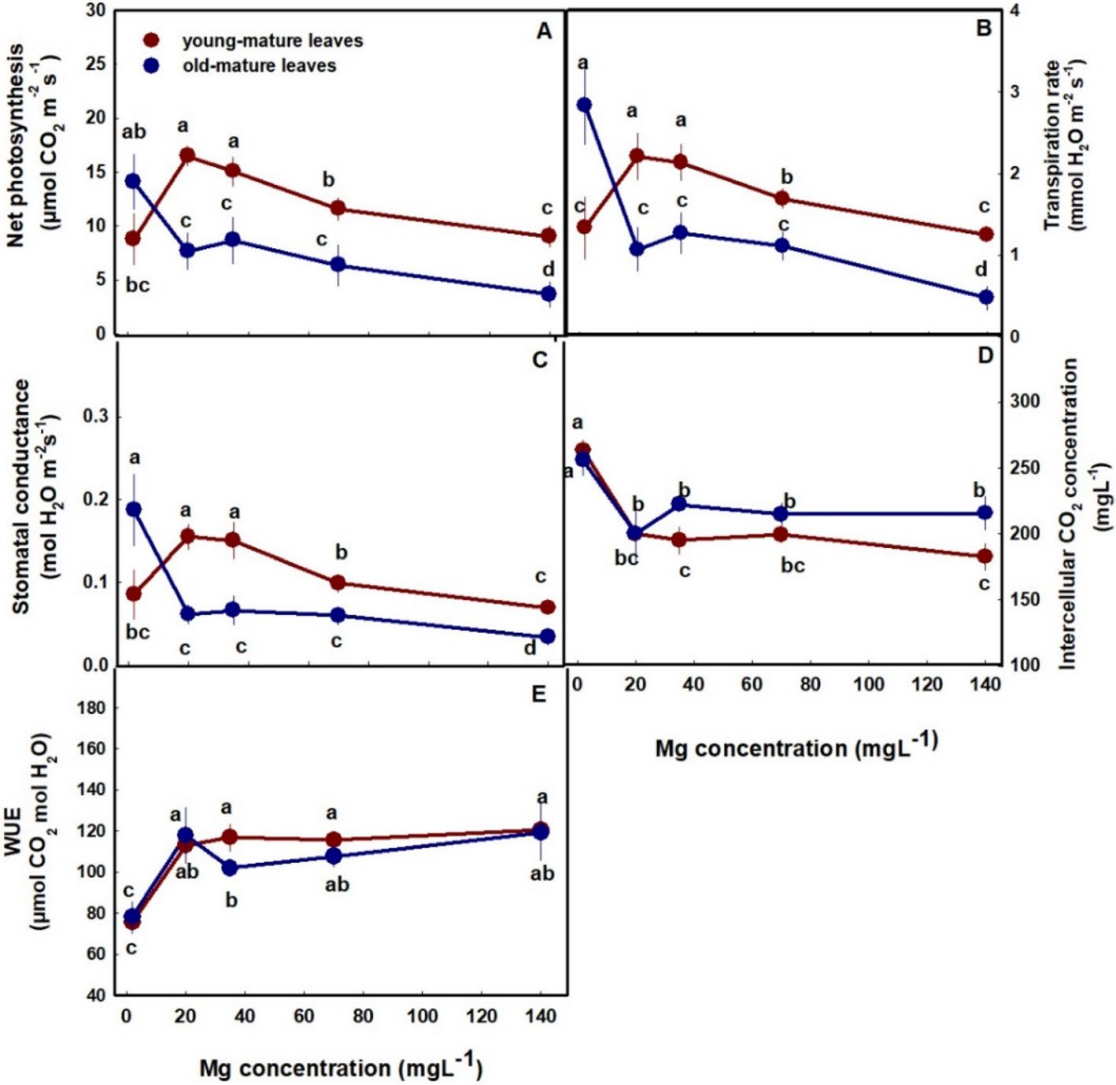
Effect of Mg concentration on gas exchange parameters in young-mature and old-mature leaves of cannabis, at the reproductive growth phase. Net photosynthesis rate (**A**), transpiration rate (**B**), stomatal conductance (**C**), intercellular CO_2_ concentration (**D**) and WUE (**E**). Presented data are averages ± SE (*n* = 6). Different small letters above the means represent significant differences between treatments by Tukey HSD test at α = 0.05

Photosynthesis and gas exchange parameters of young-mature and old-mature leaves were affected as well by Mg supply (Fig. 4A-E). In parallel with effects on the photosynthetic pigments, in young-mature leaves photosynthesis rate, transpiration rate and stomatal conduction presented optimum curves with highest values under 20–35 mg L^−1^ Mg, and reduced values under the deficiency and toxicity Mg concentrations (Fig. 4A-C). In old-mature leaves photosynthesis rate, transpiration rate and stomatal conduction were highest under the low Mg supply (2 mg L^−1^) and lowest under the highest Mg supply (140 mg L^−1^).

Internal CO_2_ concentration was highest at the deficiency treatment (2 mg L^−1^) and decreased up to a concentration of 20 mg L^−1^ in both leaves. Despite the common trend, internal CO_2_ concentration of old-mature leaves was higher than in young-mature leaves (Fig. 4D). Water Use Efficiency (WUE) was lowest under low Mg supply and increase up to a concentration of 20 mg L^−1^ in both leaves (Fig. 3E).

### Cannabinoid and terpenoid profile

Medical cannabis plants are cultivated for the female inflorescences that contain a plethora of secondary metabolites of potential biological activity. Since the chemical quality and its standardization in the plants are affected by cultivation conditions, we examined the concentrations and distribution of the inflorescences’ secondary metabolites in the plant in response to Mg supply.

**Fig. 5:**
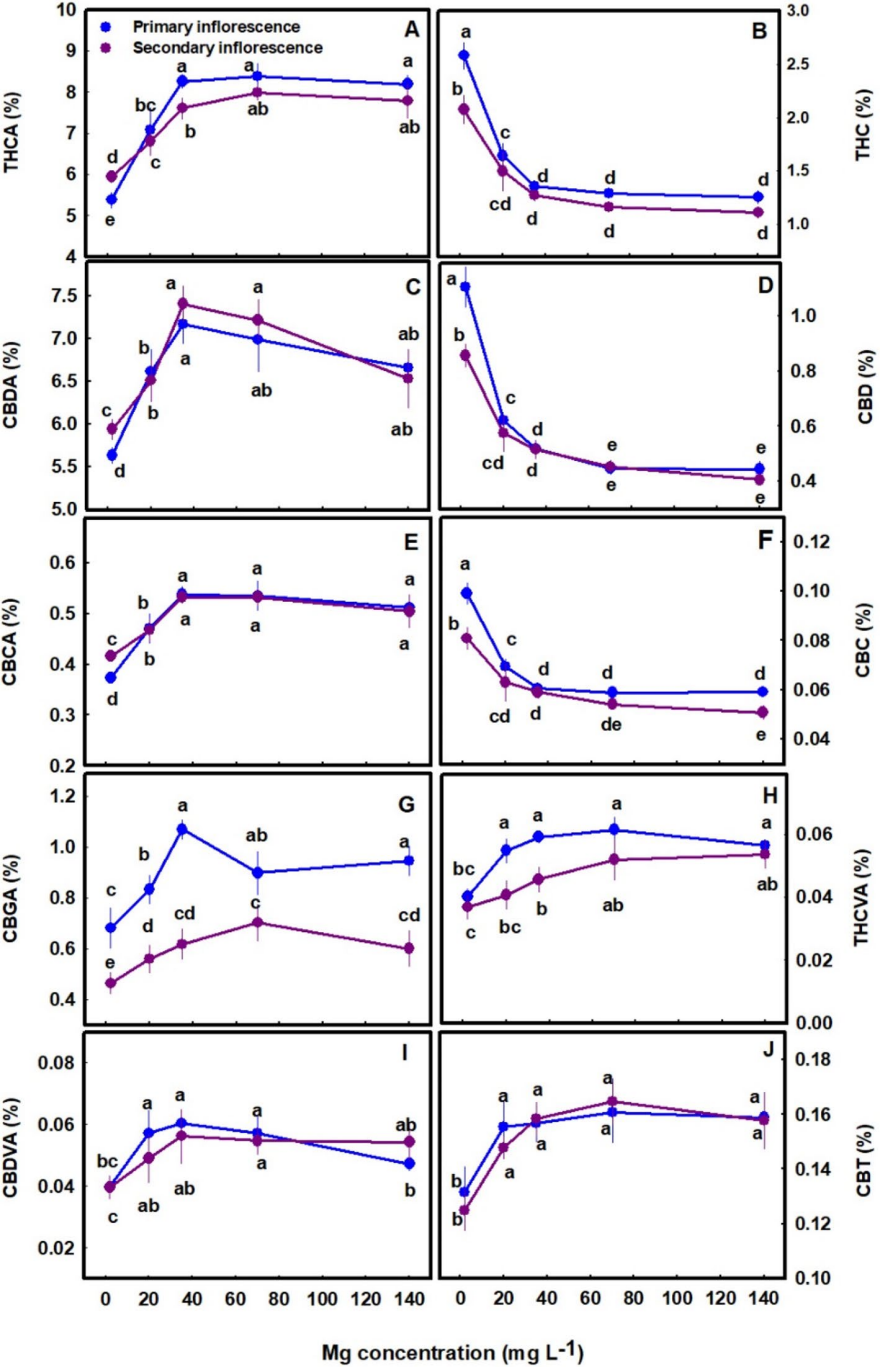
Effect of Mg application on cannabinoid concentrations in primary and secondary apical inflorescences of medical cannabis plants. ∆.^9^-tetrahydrocannabinolic acid (**A**, **B**), cannabidiolic acid (**C**, **D**), cannabichromenic acid (**E**, **F**), cannabigerolic acid (**G**), tetrahydrocannabivarinic acid (**H**), cannabidivarinic acid (**I**) and cannabitriol (**J**). The presented data are averages ± SD (*n* = 6). Different small letters above the means represent significant differences between treatments by Tukey HSD test at α = 0.05

**Fig. 6:**
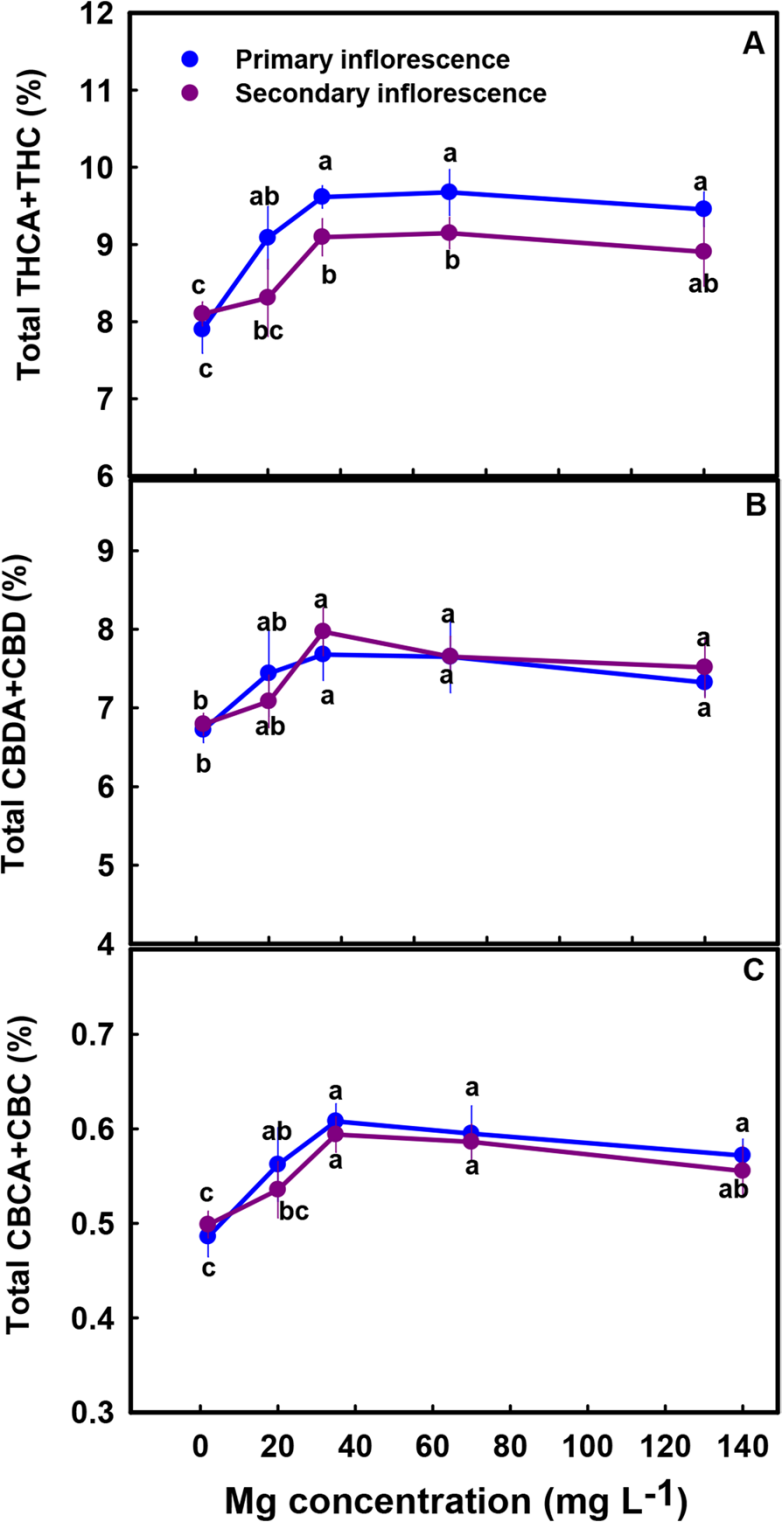
Effect of Mg application on total THC, CBD and CBC concentrations in primary and secondary apical inflorescences of medical cannabis plants. Total THC (THCA + THC) (**A**), Total CBD (CBDA + CBD) (**C**), and total CBC (CBCA + CBC) (**E**). Presented data are averages ± SD (*n* = 6). Different letters above the means represent significant differences between treatments by Tukey HSD test at *α* = 0.05

**Fig. 7:**
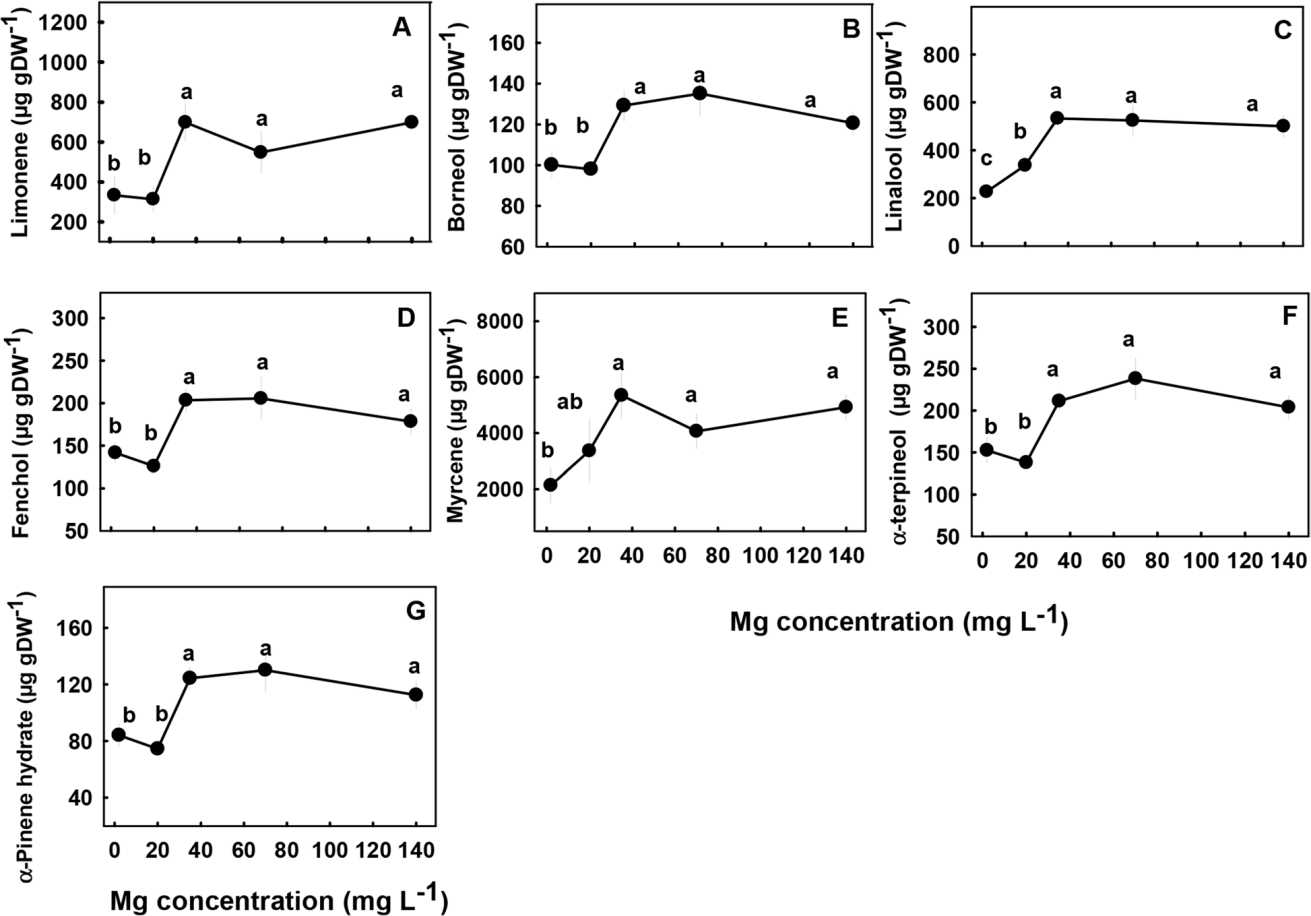
Effect of Mg supply on monoterpene concentrations in the apical inflorescence of medical cannabis plants. Limonene (**A**), borneol (**B**), linalool (**C**), fenchol (**D**), myrcene (**E**), α-terpineol (**F**), α-pinene hydrate (**G**). Data are means ± SE (*n* = 5). Different letters above the means signify significant differences by Tukey HSD test at α = 0.05

**Fig. 8:**
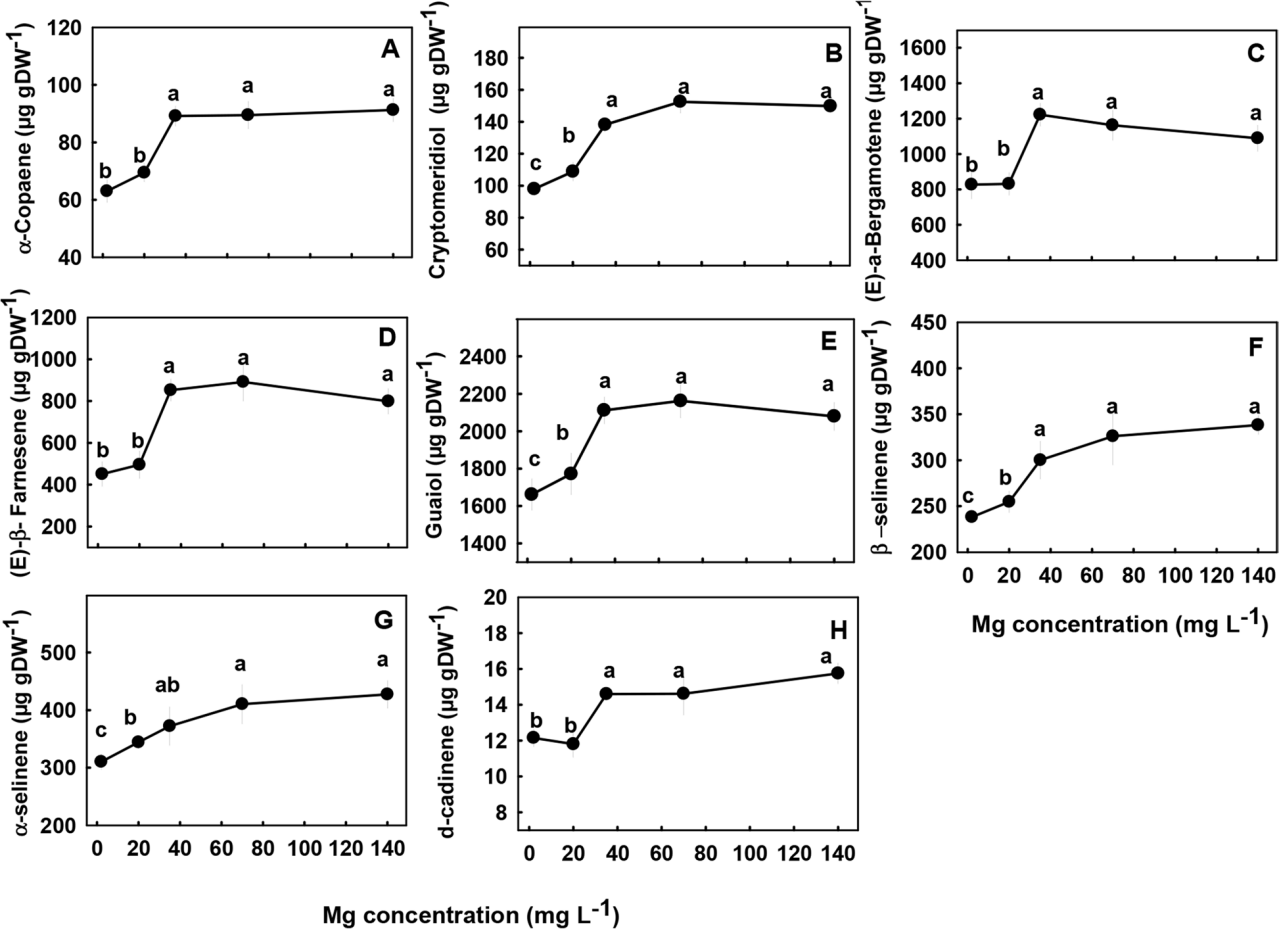
Effect of Mg supply on sesquiterpene concentrations in the apical inflorescence of medical cannabis plants. α-copaene (**A**), cryptomeridiol (**B**), **(E**)-a-bergamotene (**C**), β-selinene (**E**)-β-farnesene (**D**), guaiol (**E**), β-selinene (**F**), α-selinene (**G**), and δ-cadinene (**H**). Data are means ± SE (*n* = 5). Different letters above the means signify significant differences between treatments by Tukey HSD test at α = 0.05

The secondary metabolites profile was significantly affected by the Mg treatments (Figs. 5, 7 and 8). The cannabinoid profile in the plants revealed several trends: (i) The concentration of all the acidic (carboxylated) cannabinoids examined, including THCA, CBDA, THCVA, CBDVA, CBGA and CBCA, as well as CBT, were lowest under limited Mg supply and increased significantly with the increase in Mg supply up to the concentration of 20–35 mg L^−1^ (Fig. 5A, C, E, G-J). The same trend was also observed in the sum of the acidic and non-acidic compounds (total THC, total CBD and total CBD in the primary inflorescence was 23%, 15%, and 24.5% higher under 35 mg L^−1^ Mg supply compared to the 2 mg L^−1^ Mg supply treatment) (Fig. 6A-C) (ii) The concentration of all the non-acidic (decarboxylated) forms of the cannabinoids examined, including THC, CBD and CBC, were highest at the 2 mg L^−1^ Mg deficiency treatment and decreased with the increase in Mg supply up to the concentration rang of 35–70 mg L^−1^ Mg (Fig. 5B, D, F).

The cannabinoid concentrations were tested in two types of inflorescences: the apical (primary) inflorescence from the top of the main stem, and the apical (secondary) inflorescences of a lower branch. For most cannabinoids no significant differences were found between the concentrations in the two inflorescences, except that for several cannabinoids higher concentrations were present in the secondary inflorescences under deficiency conditions (2 mg L^−1^ Mg) (Fig. 5A, C, E). CBGA concentrations were significantly different between the two inflorescences throughout the Mg concentration range tested (Fig. 5G).

Interestingly, Mg nutrition had a considerable effect on the extent of decarboxylation, which was highest under the lowest Mg treatment, and reduced with the increase in Mg supply. The decarboxylation of THCA, CBDA and CBCA was higher by 18.28%, 9.65% and 10.84%, respectively under the deficiency treatment of 2 mg L^−1^ Mg compared to the 35 mg L^−1^ Mg treatment. Thus, a 2.26-fold averaged decrease in the decarboxylation of the acidic forms occurred as a response to the increase in Mg concentration from 2 mg L^−1^ to 35 mg L^−1^, which demonstrates a stimulation of decarboxylation by Mg deficiencies (Fig. 5A-F).

The concentrations of most terpenoids detected in the inflorescences were significantly influenced by Mg supply (Figs. 7 and 8). We identified three major trends in the response of monoterpenes and sesquiterpenes the Mg treatments; (i) Concentrations of the monoterpenes limonene, borneol, fenchol, myrcene, α-terpineol, α-pinene and the sesquiterpenes α-copaene, (E)-a-bergamotene, (E)-β-farnesene, and δ-cadinene were lowest under the low Mg supply (2–20 mg L^−1^), and increased with the increase in Mg concentration up to 35 mg L^−1^ Mg. There were no significant differences in concentrations of the terpenes listed above at the concentrations range 2–20 mg L^−1^ (Figs. 7 and 8). (ii) The monoterpene linalool and the sesquiterpenes cryptomeridiol, Guaiol, β-selinene and α-selinene had the lowest concentrations under the lowest Mg supply and increased with the increased in Mg levels up to a concentration of 35 mg L^−1^ Mg (Figs. 7C, 8B, E–G). (iii) The monoterpenes α- pinene, β-pinene, camphene and the sesquiterpenes α-humulene, selina-3,7(11)-diene, α-eudesmol, β caryophyllene, and β-eudesmol were not significantly affected by the Mg treatments (Fig. S1 supplemental).

### Macro and micronutrient concentrations

**Fig. 9:**
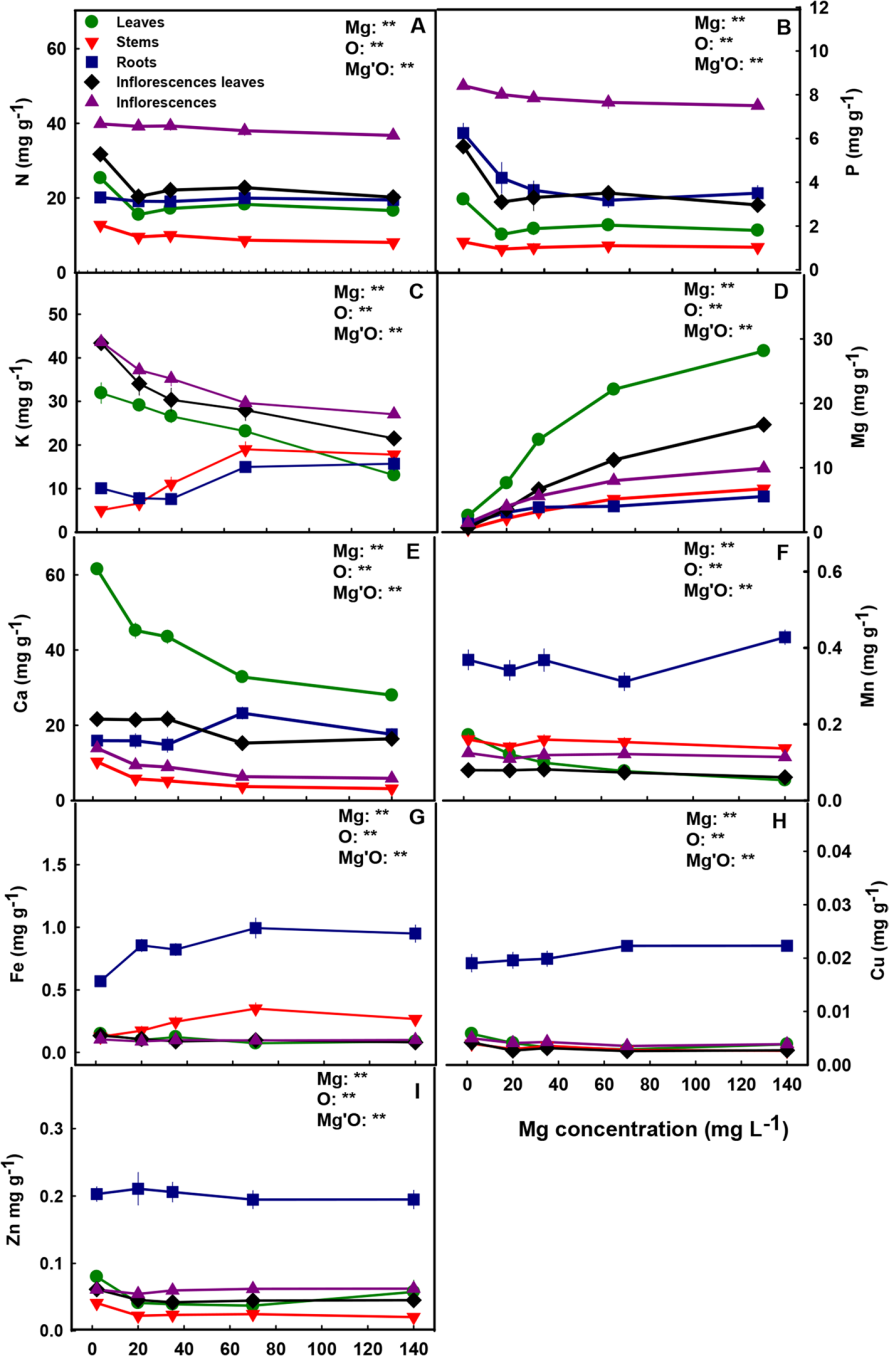
Effect of Mg supply on macro- and micronutrient concentrations in leaves, stems, roots, inflorescence-leaves and inflorescences of medical cannabis plants. Total N (**A**), P (**B**), K (**C**), Mg (**D**), Ca (**E**), Mn (**F**), Fe (**G**), Cu (**H**), and Zn (**I**). Presented data are averages ± SE (*n* = 6). Results of two-way ANOVA indicated as ** *p* < 0.05, F-test; NS, not significant *p* > 0.05, F-test. In the ANOVA results Mg is magnesium, O is organ, and Mg’O represents the interaction between Mg and O

The distribution of mineral nutrients in the plant demonstrated nutrient specificity. Ca and Mg concentrations were highest in the leaves, and N, P, and K accumulated to the highest concentrations in the inflorescences (Fig. 9). The level of Mg supplied to the plants affected the uptake of Mg into the root and its translocation to the shoot, as is evident by the increase in Mg concentration in all plant organs with the increase in Mg supply throughout the concentration range tested (Fig. 9D). Ca showed an opposite trend, and accumulated in leaves, stems and inflorescence to the highest concentration under the low Mg supply (2 mg L^−1^ Mg) and decreased with further increase in Mg supply. Ca concentration in the inflorescence-leaves was lowest under the concentration range of 70–140 mg L^−1^ Mg, while in the roots it was highest under these concentrations (Fig. 9E).

Similar to Ca, K concentration in the roots increased with the increase in Mg supply levels > 35 mg L^−1^ (Fig. 9C), which demonstrates root-accumulation of the two cations under high Mg concentrations. The stems as well accumulated K under high Mg supply levels (Fig. 9C). In leaves, inflorescence-leaves and inflorescences K concentrations were highest at the lowest Mg supply (2 mg L^−1^) and decreased with the increase in Mg supply up to 140 mg L^−1^.

N and P showed a similar accumulation trend in the plant. Their concentrations in leaves, inflorescence-leaves and stems were highest under limited Mg supply (2 mg L^−1^ Mg). In the roots, P concentration decreased with the increase in Mg supply up to the concentration of 140 mg L^−1^ Mg, while N concentration in the roots was not affected by the Mg treatments. The same trend was also seen in inflorescences, N concentration in inflorescences was not affected by Mg concentrations, while P slightly decreased with the increase in Mg supply (Fig. 9A-B).

The level of Mg supplied to the plants affected also the accumulation and uptake of micronutrients. The concentration of all micronutrients tested (Mn, Fe, Cu, Zn), was higher in the root compared to the shoot (Fig. 9F-I). The concentrations of the micronutrients in the inflorescence were usually not affected by Mg supply. In the leaves, the concentrations of the micronutrients were highest under low Mg supply (2 mg L^−1^) and decreased with the increase in Mg supply. Zn and Cu concentrations in the stems were highest under low Mg supply (Fig. 9H, I), stem’s Fe concentration increased with the increase in Mg supply up to 70 mg L^−1^ (Fig. 9G), while stem’s Mn was not affected by the Mg treatments (Fig. 9F). Mn, Fe and Cu concentrations in the root were highest under 140 mg L^−1^ Mg (Fig. 9F-H), while root’s Zn was not affected by Mg nutrition (Fig. 9I). Cu, Zn and Fe concentrations in inflorescence-leaves were highest under low Mg supply and decreased with further increase in Mg up to 35 mg L^−1^ Mg, and Mn concentration in the inflorescence leaves was not affected by Mg nutrition.

## Discussion

The present study examined the nutritional requirements of the *C. sativa* plant for Mg at the reproductive growth phase as related to plant function, development, and production of the biologically active secondary metabolites cannabinoids and terpenoids. The results revealed high sensitivity of the secondary metabolite profile to Mg nutrition, and a considerable effect of Mg availability on mineral accumulation, plant morphology and production, and gas exchange parameters, thus supporting the hypothesis. Therefore, an optimal Mg concentration is critical for obtaining optimal yield quantity and quality in cannabis plants.

### Plant visual appearance, development, and function

Plants exposed to Mg starvation (2 mg L^−1^) developed visual symptoms, and had low morpho-physiological function as is typical of Mg-deficient plants (Cakmak and Yazici 2010) (Fig. 1). Plants supplied with a low Mg dosage (2 mg L^−1^) were significantly smaller (Fig. 2A), and developed interveinal chlorosis in leaves, and small inflorescences with scorched and dead inflorescence-leaves (Fig. 1F, K). As a result, inflorescences biomass production was significantly decreased by 37.7% (compared to plants that received 35 mg L^−1^ Mg) (Fig. 2D). These results are in accord with response of cannabis plants to Mg deficiency at the vegetative growth phase (Morad and Bernstein 2023), as well as responses of other plant species that demonstrated similar visual deficiency symptoms and growth retardation under Mg-deficiency (Uchida and Silva 2000; Guo et al. 2016). Plant development at the reproductive growth phase were found to be more sensitive to Mg deficiency compared to the vegetative growth phase, as the number of nodes on the main stem was reduced under Mg supply at the reproductive (Fig. 2B) but not the vegetative stage (Morad and Bernstein 2023). This heightened sensitivity to Mg deficiency at the reproductive phase likely reflects the higher requirement for Mg to support the intensive reproductive tissue production at this stage.

Under Mg-deficiency, leaves from the bottom of the cannabis plants, that are less exposed to light, showed no signs of deficiency and remained green (Fig. 1A). This phenomenon was reported also for Mg-deficient bean plants, that showed no signs of chlorosis in shaded leaves (Marschner and Cakmak 1989; Cakmak and Kirkby 2008). The tissue damages that develop under high light intensity in Mg-deficient leaves are considered to result from oxidation damages, which are caused by enhanced generation of reactive oxygen species in the chloroplasts, at the expense of energy utilization for photosynthesis. In the active young-mature leaves of the Mg-deficient cannabis plants, photosynthesis rate was indeed lower then under optimal Mg-supply levels (Fig. 4A), and so was the rate of transpiration (Fig. 4B), and accordingly internal CO_2_ concentration was higher (Fig. 4D). The tissue damages under Mg-deficiency in these leaves is evidenced also by the higher leakage of electrolytes from the membrane (Fig. 3A), which is known to results from oxidative-stress induced lipid peroxidation (Bernstein et al. 2010). The reduced photosynthesis rate under the Mg-deficiency conditions can be attributed to a direct effect of Mg-deficiency to suppress activity of enzymes involved in CO_2_ fixation (Tränkner et al. 2018), but also to the reduced concentrations of the main photosynthetic pigments (chlorophylls) (Fig. 3), which require Mg for their biosynthesis as well as a structural element (Hawkesford et al. 2012). Similar reduction in photosynthetic pigment concentrations and photosynthesis rate were apparent also in leaves of Mg-deficient cannabis plants at the vegetative stage of development which demonstrated as well similar visual deficiency symptoms (Morad and Bernstein 2023).

The restricted Mg availability affected also water relation of the tissue, as relative water content was lower under limited Mg supply (Fig. 3C). This is in accord with previous reports on effects of Mg supply on water relations in the tissue which demonstrated a reduction in transpiration rate under Mg deficiency (Jezek et al. 2015; Peng 2015), that might be related also to the involvement of Mg in regulation of stomatal opening (Inoue et al. 2022).

Mg toxicity symptoms are rarely described to occur in plants, and no reports are available for direct effect of excessive Mg supply on plant metabolism (Gerendás and Führs 2013; Verbruggen and Hermans 2013). One possible reason for the scarcity of Mg toxicity reports could be the large vacuolar storage capacity for Mg within the plant, which prevents Mg build-up to damaging concentrations in the cytoplasm (Hawkesford et al. 2012; Chen et al. 2018). Compartmentation of Mg in the vacuole under high Mg supply was fond to be regulated by dedicated Mg transporters, and is considered to play a role also in charge compensation and osmoregulation in the vacuole (Conn et al. 2011; Chen et al. 2018).

In the cannabis plants, over-supply of Mg (70–140 mg L^−1^) did not significantly affect morphological parameters. However, a slight, yet significant, decrease in inflorescences and total plant biomass was observed (by 8.6% and 12.5%, respectively) in plants that received 140 mg L^−1^ Mg compared to 35 mg L^−1^ Mg (Fig. 2D). This effect is likely related to the observed restrictive effects of Mg-oversupply on C-fixation. Photosynthetic activity in plants is often positively correlated to crop biomass accumulation (Zhu et al. 2010), as increased C-fixation enhances the energy status of the plant and carbohydrate availability, and hence facilitates growth stimulation. In our cannabis plants as well, net photosynthesis, transpiration rate and stomatal conductance decreased under over-supply of Mg (Fig. 4A-C) in both the young-mature and old-mature leaves, likely resulting in the reduced inflorescence biomass production. These toxicity effects were accompanied by development of visual toxicity symptoms e.g., burnt leaf tips followed by leaf chlorosis and burnt inflorescence-leaves tips (Figs. 1E, J, O), and the tissue damage was apparent as well by the increase in electrolyte leakage from old-mature leaves, and the reduced pigment concentrations (Figs. 3A, D, E).

Tissue toxicity damages were more pronounced in old leaves, which is likely related to a potential higher accumulation of Mg in these leaves. Unlike at the reproductive growth phase, during vegetative growth, gas-exchange parameters in young-mature leaves were not significantly affected by oversupply of Mg (Morad and Bernstein 2023), and accordingly no reduction in plant biomass was observed. The lower sensitivity to excess Mg at the vegetative growth phase probably reflects the high demand for Mg of the intensely-growing vegetative tissues (especially leaves), for example as structural component of the chlorophyll molecule; while at late stages of reproductive growth when growth is reduced so does the utilization of Mg, resulting in increased accumulation in the tissues. This is supported by the considerably higher concentrations of Mg in the leaves at the reproductive compared to the vegetative growth phase, i.e., 30 vs. 18 mg g^−1^, respectively, under the highest Mg supply treatment (Fig. 9D; Morad and Bernstein 2023).

### Cannabinoids and terpenes profile

Biosynthesis of secondary metabolites is well known to be affected by exogenous factors including cultivation conditions (Verpoorte et al. 2002; Ramakrishna and Ravishankar 2011; Gorelick and Bernstein 2014). Since the biological activity of cannabis is based on its’ secondary metabolite profile, understanding the impact of Mg nutrition on the medical cannabis crop, requires knowhow of the impact on plant secondary metabolism. In the current study, we revealed that Mg nutrition elicits considerable changes to cannabinoid and terpene concentrations in cannabis plants (Figs. 5, 7–8). The dose response trend for Mg input was similar for the acidic (carboxylated) cannabinoids (i.e., THCA, CBDA, THCVA, CBDVA, CBGA, CBCA) and most terpenes. Both compound groups showed low concentrations under Mg deficiency conditions (2–20 mg L^−1^ Mg) which increased with the increase of Mg supply to the plants, suggesting an optimal Mg application at the concentration of 35 mg L^−1^ Mg.

It is not surprising that cannabinoid and terpene production was reduced in response to a limited Mg supply, owing to Mg requirement for the activity of several key enzymes in the biosynthesis pathway of both groups of compounds (Eisenreich et al. 2001; Degenhardt et al. 2017). Furthermore, Mg is required also for the formation of Geranyl diphosphate (GPP) that participates in the biosynthesis of both terpenes and cannabinoids (Degenhardt et al. 2017).

Terpenes are a structurally diverse group of natural compounds that are synthesized from IPP, which is formed in the Mevalonate pathway and the Deoxyxylulose pathway, and from its allylic isomer DMAPP (Eisenreich et al. 2001; Flores-Sanchez and Verpoorte 2008). Cannabinoids are prenylated polyketides of mixed biosynthetic origin that are synthesized from fatty acid and isoprenoid precursors (Stout et al. 2012). They are synthesized from two pathways: the Deoxyxylulose pathway that produces GPP and the Polyketide pathway that produces olivetolic acid (OA). GPP and OA are condensed by the GOT enzyme to form CBGA, the central precursor for cannabinoid biosynthesis (Flores-Sanchez and Verpoorte 2008). The first reaction in the biosynthesis of isoprenoid (i.e., Deoxyxylulose pathway) is the conversion of D-glyceraldehyde 3-phosphate and pyruvate into 1-deoxy-D-xylulose 5-phosphate, by the enzyme 1-Deoxy-D-xylulose 5-phosphate synthase (DXS) followed by 1-Deoxy-D-xylulose 5-phosphate reductoisomerase (DXR) (Lichtenthaler 1999). These enzymes were found to require Mg and Mn for activity (Yajima et al. 2007; Gräwert et al. 2011). Furthermore, also 4-Diphosphocytidyl-2C-methyl-D-erythritol synthase (IspD) that is essential for IPP biosynthesis (Kuzuyama et al. 2000; Rohdich et al. 2000) requires Mg for activity (Eisenreich et al. 2001). Moreover, Mg is known as a cofactor in the biosynthesis of isoprenoid in tobacco (Facchini and Chappell 1992) and natural rubber (Cornish 2001), and was also reported to bind to the diphosphate part of GPP (Ito et al. 1992). Therefore, a direct connection between plant Mg status and the production of GPP is expected, and can explain the common reduction trend of cannabinoids and terpenoids which we identified under Mg deficiency.

Two other enzymes that are involved in cannabinoid biosynthesis and require Mg for their activity are hexanoyl-CoA synthetase (CsHCS1), and GOT. The *C. Sativa* CsHCS1 enzyme, which is considered to be a trichome-specific enzyme, was reported to produce hexanoyl-CoA using hexanoate and CoA as substrates, an important step in the biosynthetic pathway of olivetolic acid in the polyketide pathway (Degenhardt et al. 2017). Based on transcript levels, CsHCS1 is the most abundant acyl-activating enzyme (AAE) in trichomes and it requires divalent cations for activity with a preference to Mg (Stout et al. 2012; Degenhardt et al. 2017). GOT is the first enzyme in the biosynthesis of cannabinoids that catalyzes the first step in cannabinoid formation, namely condensing GPP and OA for the production of CBGA (Flores-Sanchez and Verpoorte 2008). Not only that the enzyme reaction was clearly shown to be Mg dependent, the omission of GPP or olivetolic acid leads to no reaction products (Fellermeier and Zenk 1998). This is evidenced also by the low concentrations of CBGA production under Mg deficiencies in our study (Fig. 5G) as well as by the low concentrations of its products THCA, CBDA and CBCA (Figs. 5A, C, E).

When plants are exposed to stress, particularly mineral-nutrition stress, the production of secondary metabolites may increase because photosynthesis is often less inhibited then growth processes, and the excess fixed carbon is therefore allocated to secondary metabolite production (Ramakrishna and Ravishankar 2011). This was demonstrated for numerous plant species (Rajendran et al. 1992; Said-Al Ahl and Abdou 2009; Wu et al. 2020), and was shown to occur also in cannabis in previous studies from ours laboratory, which identified a high concentration of cannabinoids and terpenes in response to N deficiency (Saloner and Bernstein 2021) and P deficiency (Shiponi and Bernstein 2021b). However, the effect of Mg-deficiency stress on the production of secondary metabolites in plants is diverse; in tea (*Camellia sinensis* L.) secondary metabolite production was elevated under short-term Mg deficiency but reduced under long-term Mg deficiency (30 days) (ILi et al. 2021). In *Tanacetum parthenium* Mg deficiency enhanced the proportion of monoterpenes and decreased the proportion of sesquiterpenes in the essential oil (Farzadfar et al. 2017), and in apple adding Mg to the medium tripled anthocyanin content and the highest concentration of anthocyanin was obtained at the highest Mg concentration (Zahedzadeh et al. 2015). In the current study, although there was a general increase in the concentration of most cannabinoids and terpenoids with the increase in Mg availability, the production of some of the compounds, for example, α- pinene, β-pinene, camphene, α-humulene, selina-3,7(11)-diene, β -caryophyllene, α -eudesmol and β -eudesmol were not significantly affected by the Mg treatments (Fig. S1 supplement), demonstrating some metabolite specificity in the response to the Mg stress.

In cannabis plants, the Mg deficiency stress was manifested in impaired morpho-physiological function (e.g., reduced inflorescences biomass, photosynthetic rate, plant height, and photosynthetic pigments concentrations under low Mg supply) (Figs. 2, 3 and 4), which is correlative to the low production of terpenes and acidic cannabinoids (Figs. 5, 7–8). Also, the increased decarboxylation of THCA, CBDA and CBCA under limited Mg supply (Fig. 5), indicates that a low physiological state promoted decarboxylation of cannabinoids in the plant, and an increase in Mg supply up to 35 mg L^−1^ Mg inhibited the *in-planta* decarboxylation. We therefore suggest, that the restriction of cannabinoid and terpenoid production under Mg deficiency is caused by two possible routes: (i) a direct restriction of the activity of enzymes involved in the biosynthesis of these compounds by lack of available Mg for their activation, and/or (ii) a reduction in the availability of carbohydrates in the production sites, i.e., the inflorescences, as a result of restriction of sugar translocation in the phloem under Mg-deficiency that was demonstrated for many plant species (Elkhouni et al. 2016).

The present study therefore reveals that the response of the production of secondary metabolites to mineral nutrients in cannabis (i.e., cannabinoids and terpenes) is ion specific, and for Mg, low production of secondary metabolites corresponds to deficient availability induced low morpho-physiological function.

### Interrelation between Mg supply and the cannabis plant ionome

Availability of macro and micronutrients has a large effect on plant growth, development and function and is therefore critical for agricultural productivity and yield (Fageria et al. 2010; Kirkby 2012). Nutrient uptake by plant roots is affected by interactions, synergistic or antagonistic, between nutrients, that occur in the soil, in root-uptake mechanisms and during *in-planta* transport. Mg is known to interact with other minerals for root uptake, and especially to compete with other cations and thus have the potential to induce deficiency responses (Fageria 2001; Römheld 2011).

Our results reveal that the response of the cannabis ionome to Mg nutrition at the reproductive and the vegetative phases are similar (Fig. 9D; Morad and Bernstein 2023). In both stages of development Mg concentrations increased with the increase in Mg supply in all plant organs and accumulated to the highest concentrations in the leaves and the inflorescence leaves. This high localized accumulation of Mg in leaves under over-supply of Mg suggests restriction of retranslocation of Mg to the inflorescences under oversupply of Mg. Such localized accumulation can be facilitated by activity of tonoplast transporters, such as AtMRS2-1 and AtMRS2-5 (Shaul 2002), MGT2 and MGT3 (Lenz et al. 2013) that participate in compartmentation of Mg in vacuoles of leaf mesophyll cells under high-Mg conditions to store excessive Mg and maintain cytosolic homeostasis (Shaul 2002; Chen et al. 2018).

Different levels of Mg in the nutrient solution affected the uptake and distribution of nutrients in the plant, pointing at interactions between Mg and other plant nutrients (Fig. 9). Generally, deficient levels of Mg (2 mg L^−1^ Mg) caused higher concentrations of the macronutrients N and P in all plants organs as was also apparent at the vegetative growth phase (Morad and Bernstein 2023). The highest concentration of both nutrients was found in the inflorescences (Fig. 9A, B), and this pattern of high demand for N and P in reproductive organs was shown also in previous studies of cannabis plants at the reproductive stage (Saloner and Bernstein 2021, 2022b; Shiponi and Bernstein 2021b; Westmoreland and Bugbee 2022).

Mg supply affected K concentrations in plant organs similarly in both stages of growth; in leaves, inflorescence-leaves and inflorescences K concentration decreased with the increase in Mg supply, while in the roots it increased with the increase in Mg at part of the concentration range (Fig. 9C; Morad and Bernstein 2023). This points at competitive interaction between K and Mg for root-shoot translocation as well as ability for K compartmentation in the stem and the root. For Ca as well, the dose–response trends to Mg were similar for the vegetative and the reproductive growth phases. In all the shoot organs Ca concentration decreased with the increase in Mg levels, while the accumulation in roots was highest under high Mg supply only at the reproductive stage (Fig. 9E; Morad and Bernstein 2023). The increase in root Ca accumulation under high Mg supply at the reproductive growth phase suggests competition between Ca and Mg for root-shoot translocation. An opposing response trend of both K and Ca accumulation to Mg doses has been well demonstrated for other plant species (Hermans et al. 2004; Jezek et al. 2015; Rietra et al. 2017), pointing at a competitive interaction between Mg and these cations for plant uptake.

Micronutrients play a key role in growth and development of plants (Kirkby 2012), and are well known to interact with other minerals (Hawkesford et al. 2012) and particular with Mg (Sagardoy et al. 2009; Hermans et al. 2011). Therefore, changes in micronutrient concentrations (i.e., Mn, Fe, Zn and Cu) in response to different levels of Mg supply were expected. The micronutrients tested accumulated to the highest concentrations in the roots (Figs. 9F-I), in accord with previous studies for the vegetative growth phase from our laboratory (Saloner et al. 2019; Saloner and Bernstein 2020; Shiponi and Bernstein 2021a). In addition, under Mg deficiency (2 mg L^−1^), the concentrations of all micronutrients in the leaves were highest and decreased with the increase in Mg supply. As all the micronutrients tested are cations, high accumulation under conditions of limited Mg supply likely reflects competition between cations.

Taken together, the mineral analyses results demonstrate high requirement of the reproductive organs (inflorescence) for the major macronutrients (N, P, K); compartmentation of oversupply of heavy metal micronutrients in the roots; and antagonistic interactions between Mg and other cation macronutrients for root-uptake and *in-planta* translocation. Some variations in the effect of Mg on distribution of minerals to the plant organs between the reproductive and the vegetative stages, demonstrate developmental stage dependency.

## Conclusions

The physiological results revealed a significant impairment of the morpho-physiological function of cannabis plants under the severe Mg deficiency caused by low Mg supply of 2 mg L^−1^ Mg; and damage following an over-supply of Mg was observed under the concentration range of 70–140 mg L^−1^ Mg. Under Mg deficiency and toxicity concentrations, the inflorescences biomass was significantly decreased. The secondary metabolite concentrations were lowest under the deficiency concentrations range of 2–20 mg L^−1^ Mg, which was accompanied also by low morpho-physiological function of the plants. Although a low morpho-physiological condition was observed also in Mg oversupplied plants, the production of secondary metabolites (i.e., cannabinoids and terpenes) was not affected. Taken together, we suggest that 35 mg L^−1^ Mg is within the optimal concentration range for excelled yield quantity as well as quality, i.e., high secondary metabolite production. It should be considered that mineral requirements may vary slightly between cultivars and growing conditions, and validation of the optimal Mg concentrations is recommended to be performed across various growing conditions, and multiple cultivars with differing cannabinoid/terpene profiles.

## Supplementary Information

Supplementary Material 1.

## Data Availability

All data is available in the manuscript and the supplementary material.
