# Supplementary material for: From deficiency to toxicity: Magnesium increases cannabinoid and terpene production in cannabis plants
Source: J Cannabis Res. 2025 Dec 10;7:103. doi: 10.1186/s42238-025-00358-9 (PMC12739851; doi:10.1186/s42238-025-00358-9)
Supplement: Supplementary file 1 — Supplementary Material 1. [file 42238_2025_358_MOESM1_ESM.pdf]

Supplementary data

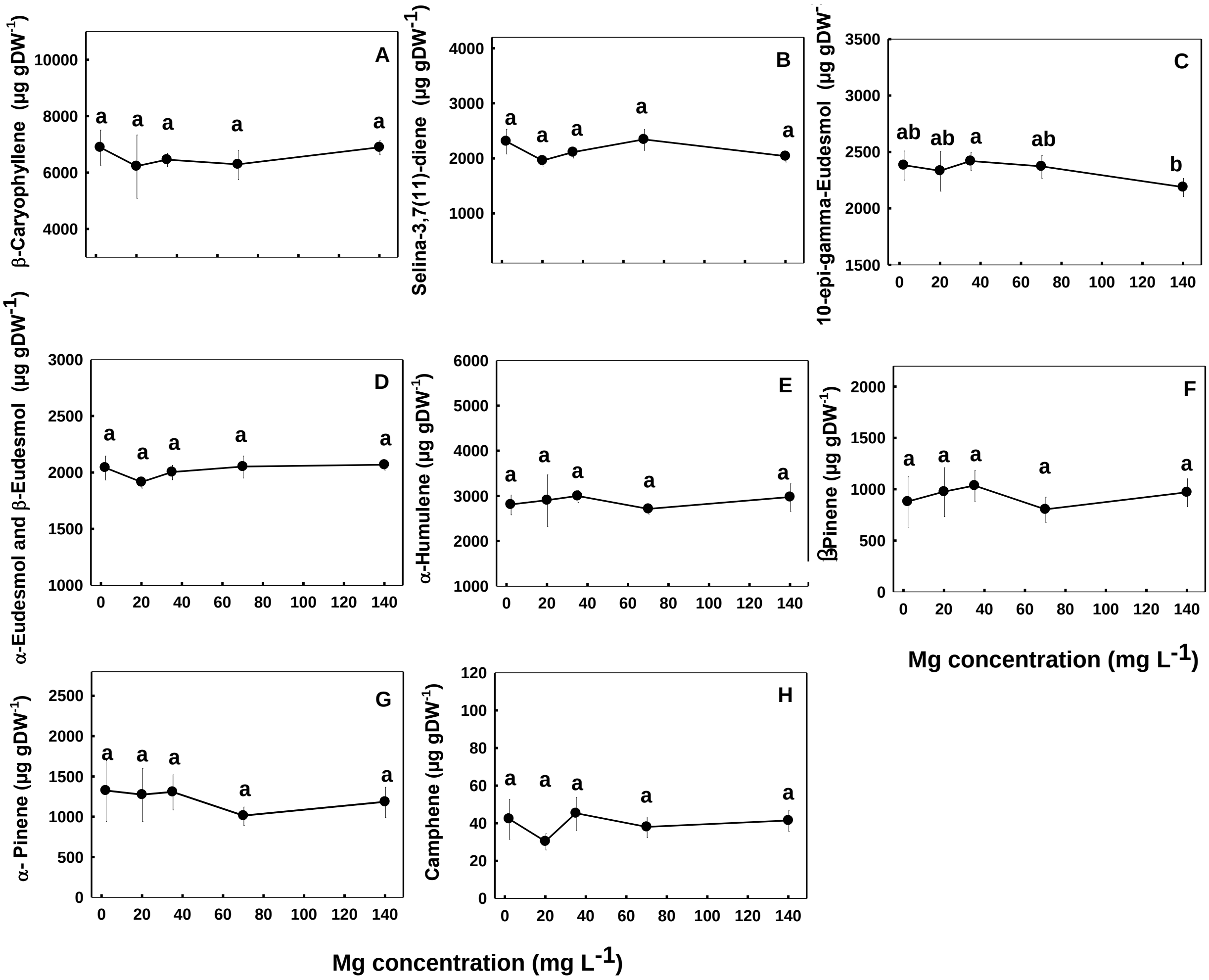

Fig. S1 supplemental: Effect of Mg supply on concentrations of additional terpenes at the apical inflorescence of medical cannabis plants. β caryophyllene (A), selina-3,7(11)-diene (B), 10-epi-γ-eudesmol (C), α-eudesmol and β-eudesmol (D), α-humulene (E), β-pinene (F), α- pinene (G), camphene (H). Data are means ± SE (n = 5). Different letters above the means signify significant differences by Tukey HSD test at *α* = 0.05.
